# Supplementary material for: Molecular generative model based on conditional variational autoencoder for de novo molecular design
Source: J Cheminform. 2018 Jul 11;10:31. doi: 10.1186/s13321-018-0286-7 (PMC6041224; doi:10.1186/s13321-018-0286-7)
Supplement: Supplementary file 1 — Supplementary material 1 (docx 791 KB) [file 13321_2018_286_MOESM1_ESM.docx]

Supporting Information

Molecular generative model based on conditional variational autoencoder for de novo molecular design

Jaechang Lim^1^, Seongok Ryu^1^, Jinwoo Kim^1^, and Woo Youn Kim^1,2^

^1^Department of Chemistry, KAIST, 291 Daehak-Ro, Yuseong-Gu, Daejeon, 34141, Republic of Korea

^2^KI for Artificial Intelligence, KAIST 291 Daehak-ro, Yuseong-gu, Daejeon, Republic of Korea


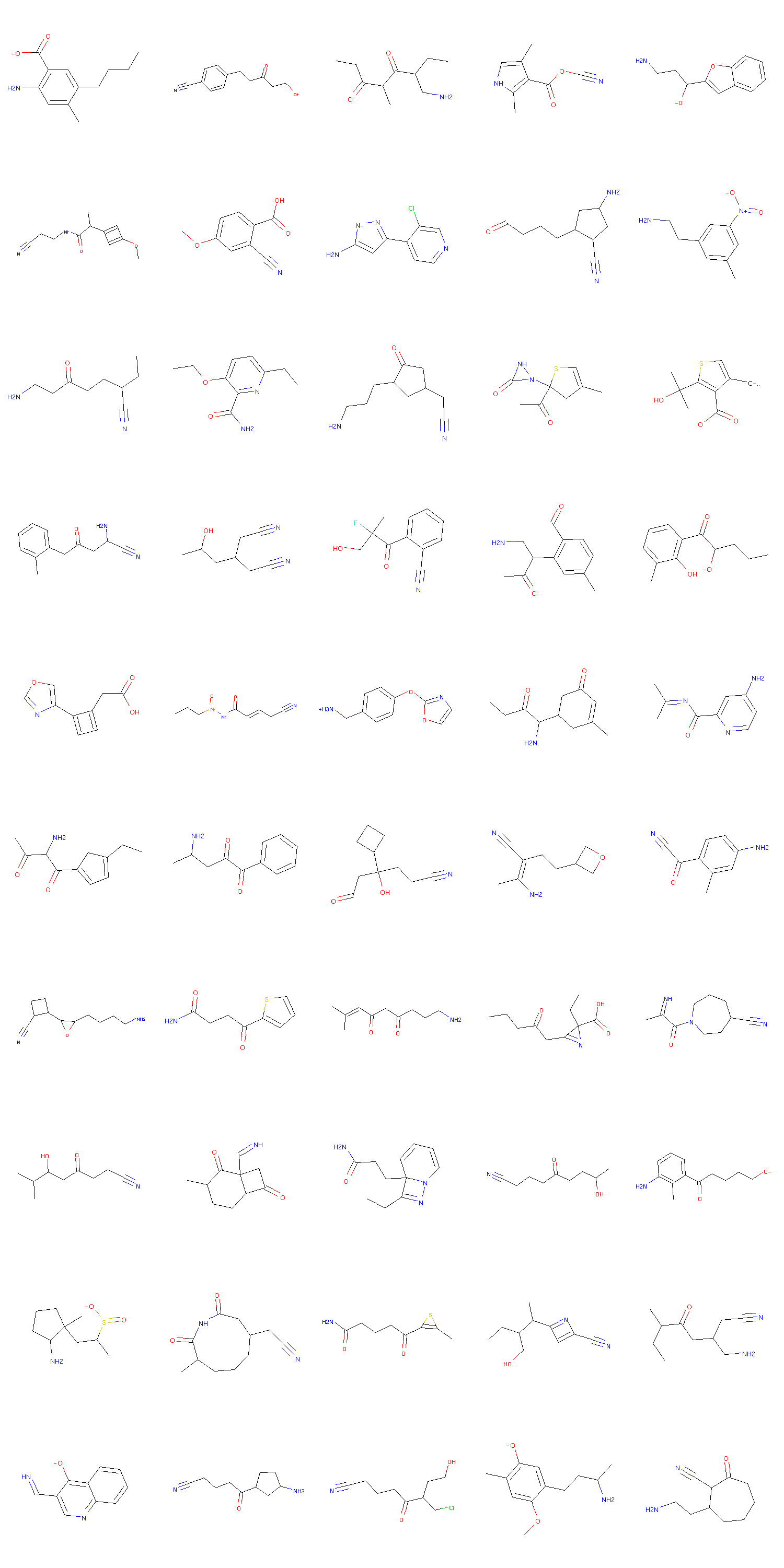

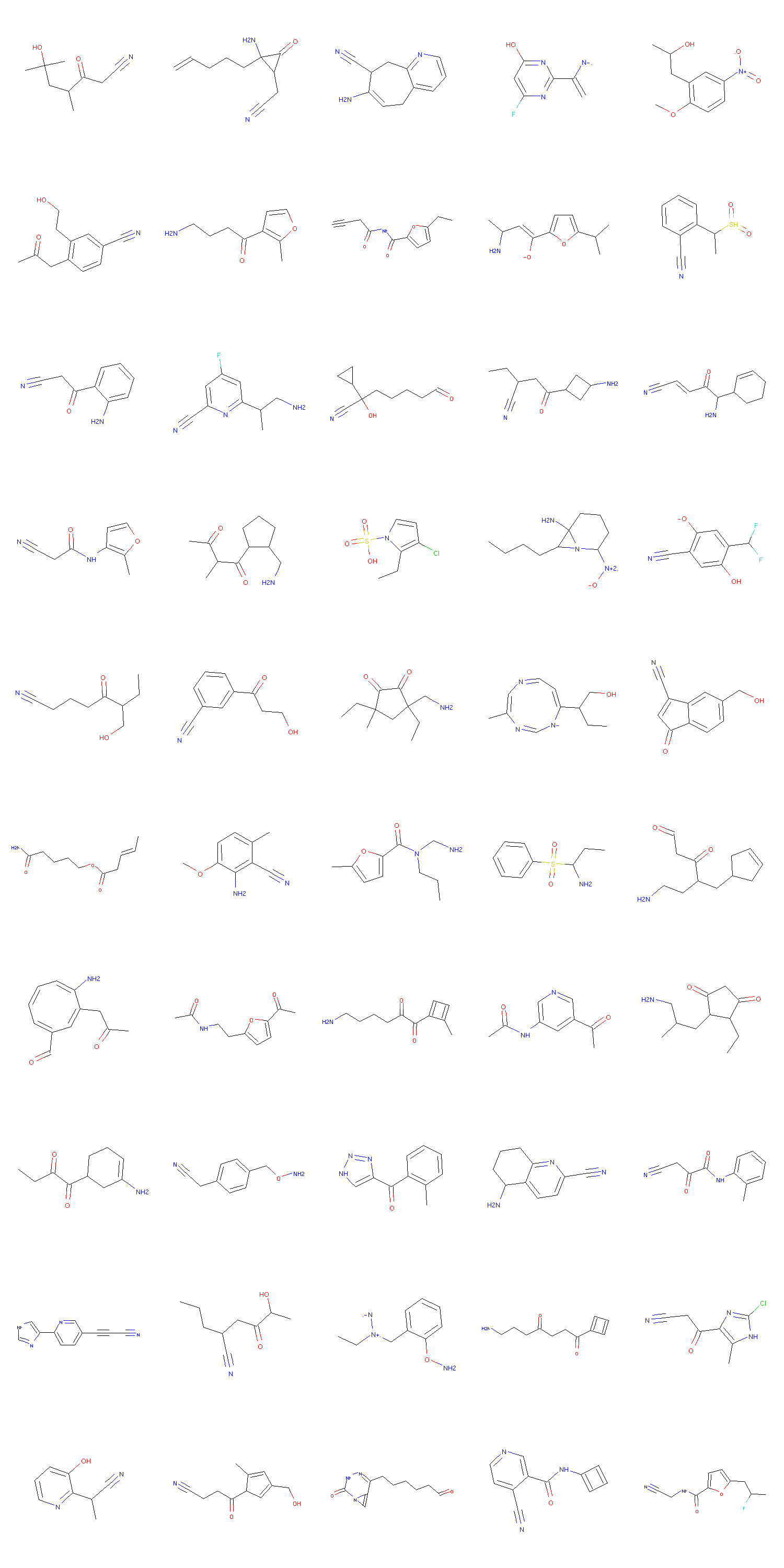


**Figure 1S**. 100 molecules whose 5 properties are same as those of Aspirin


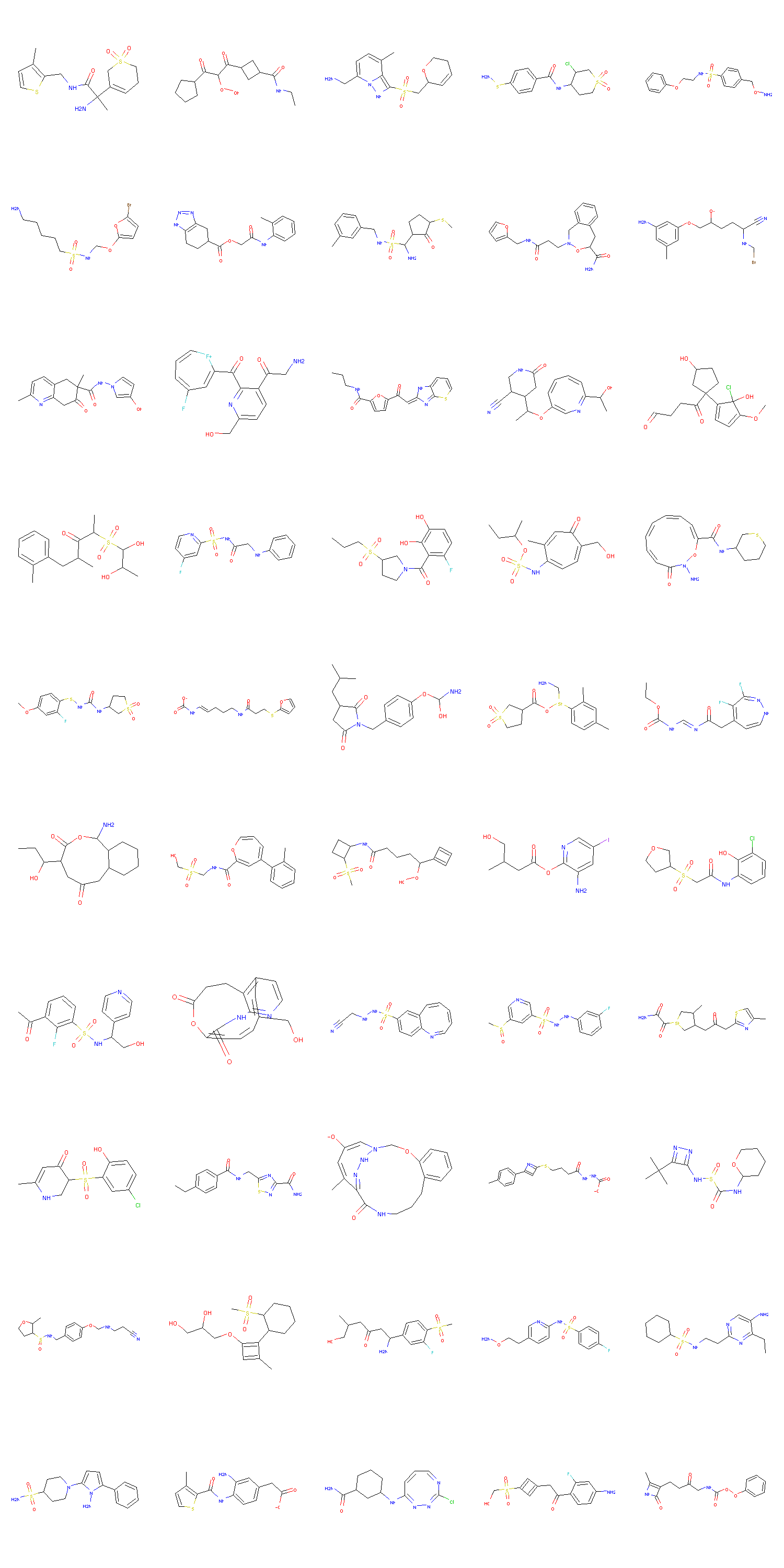

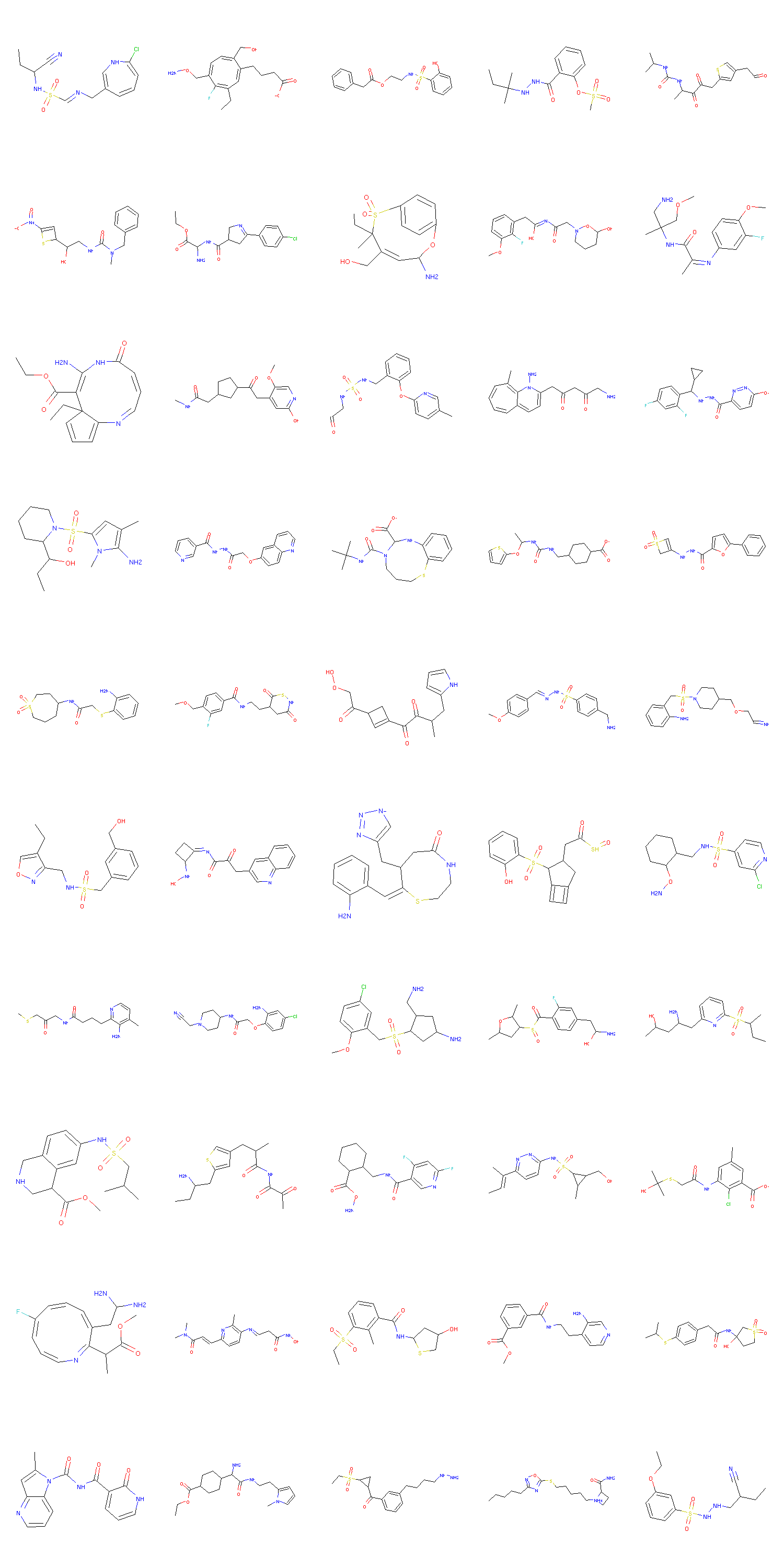


**Figure 2S**. 100 molecules whose 5 properties are same as those of Tamiflu


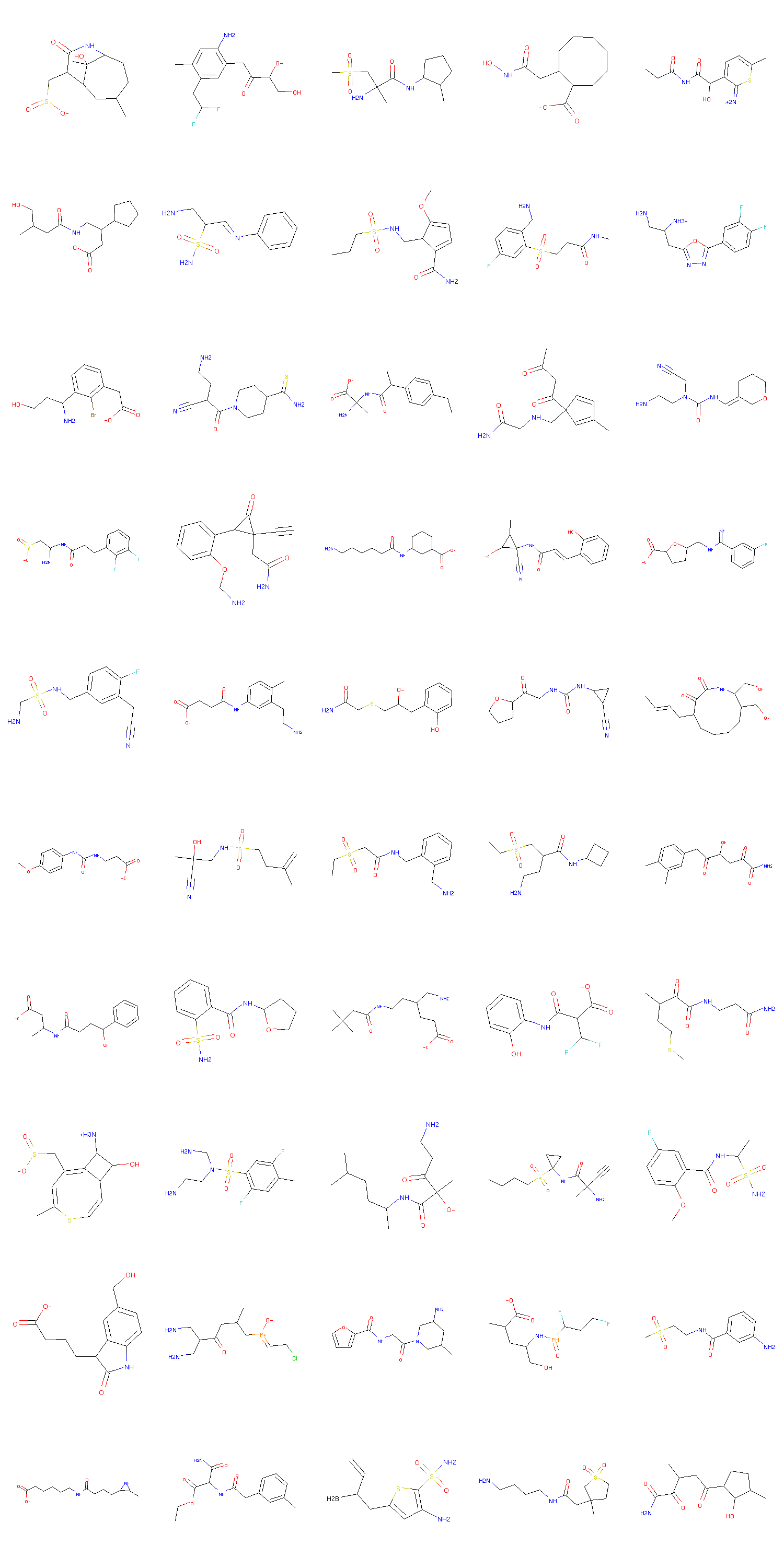

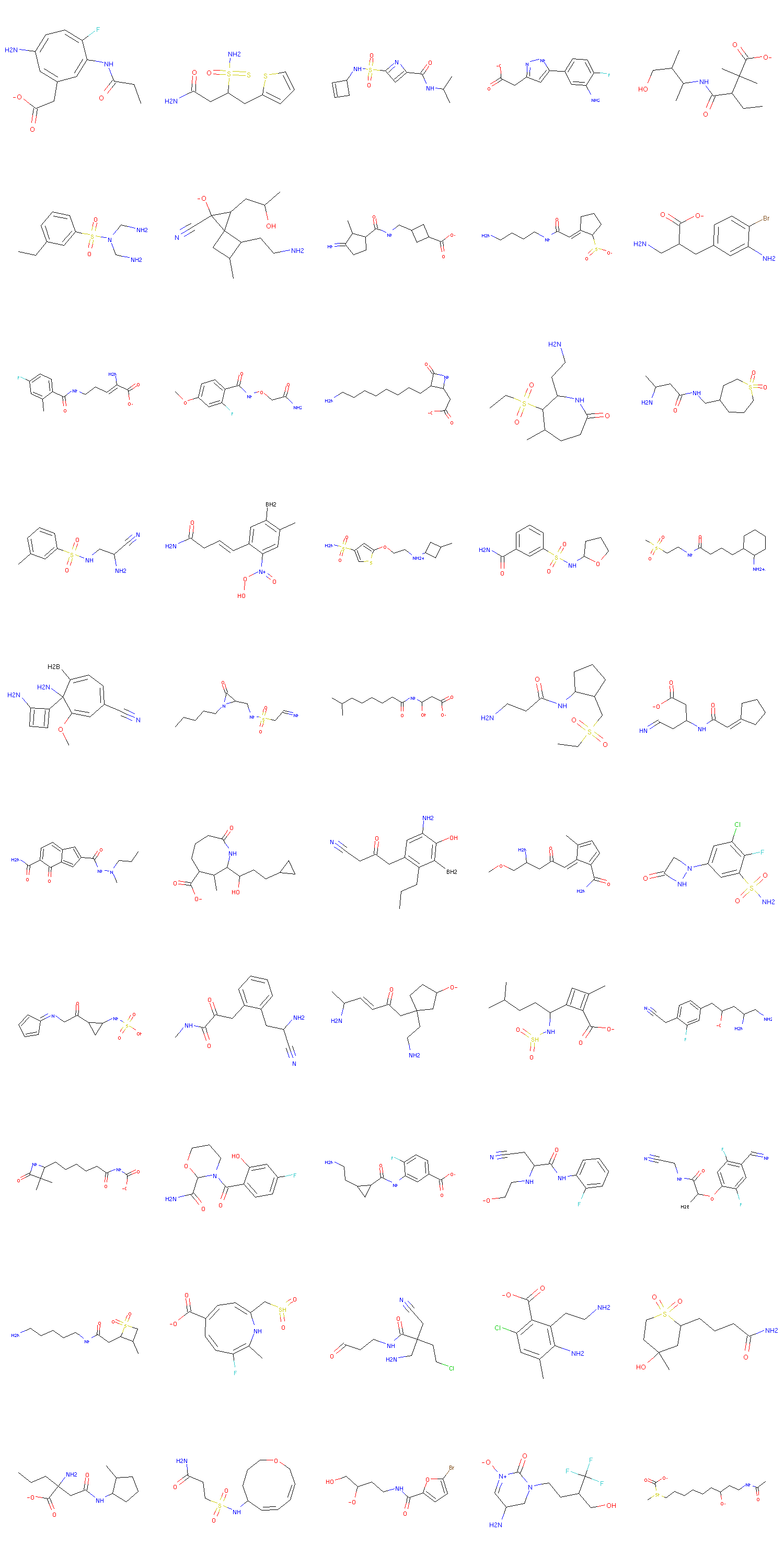


**Figure 3S**. 100 molecules whose 5 properties are same as those of Lenalidomide


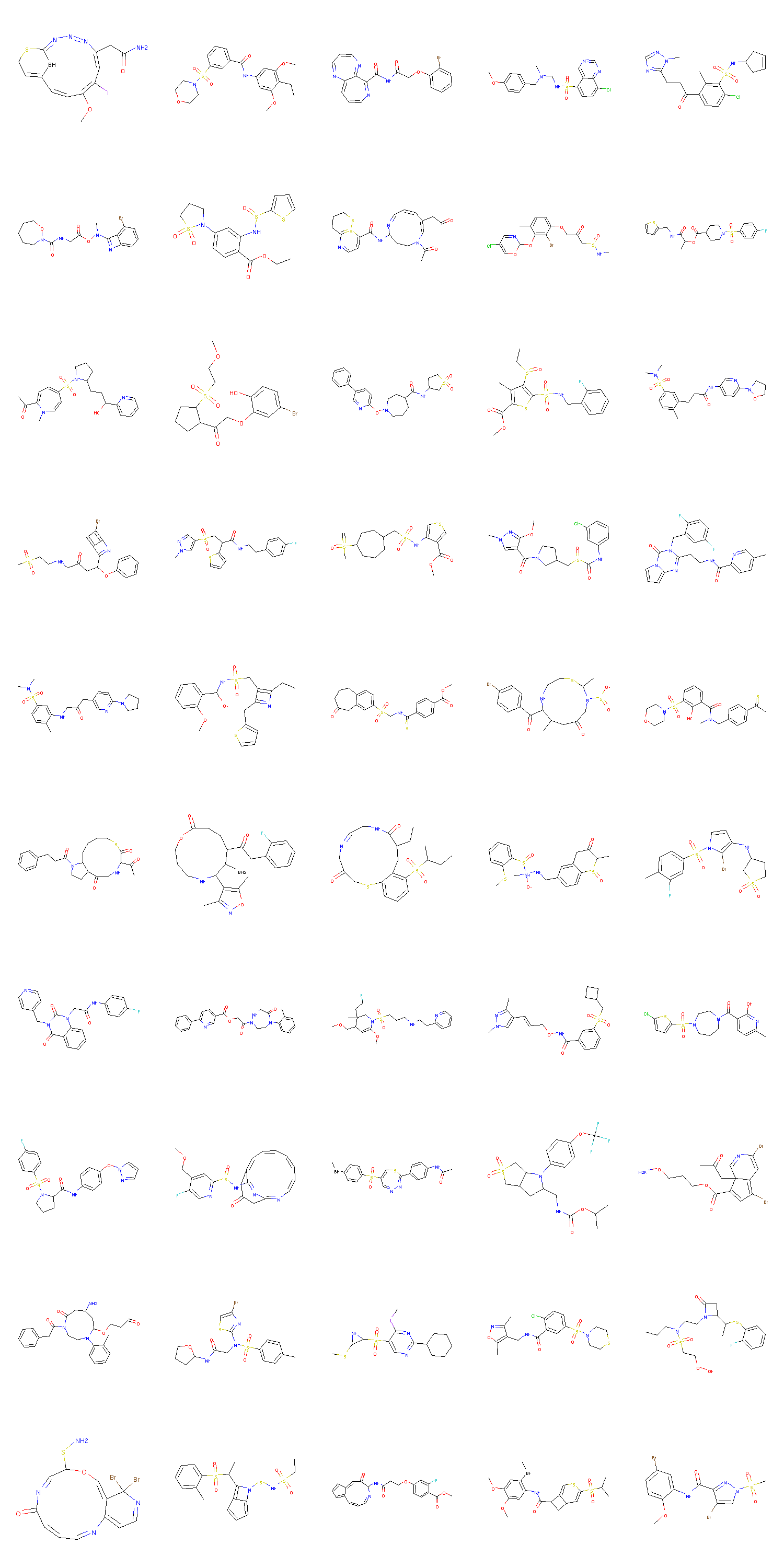

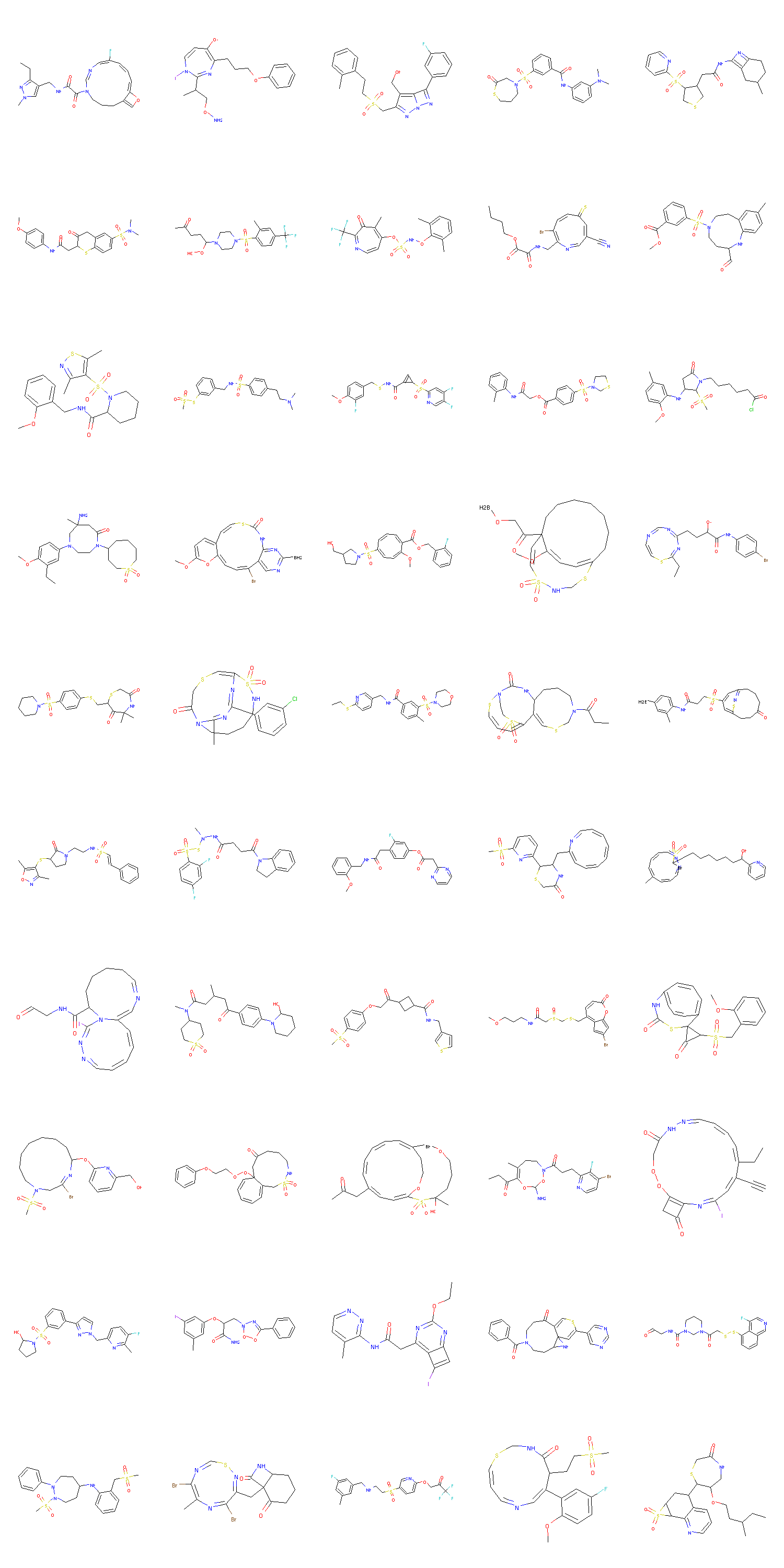


**Figure 4S**. 100 molecules whose 5 properties are same as those of Rivaroxaban


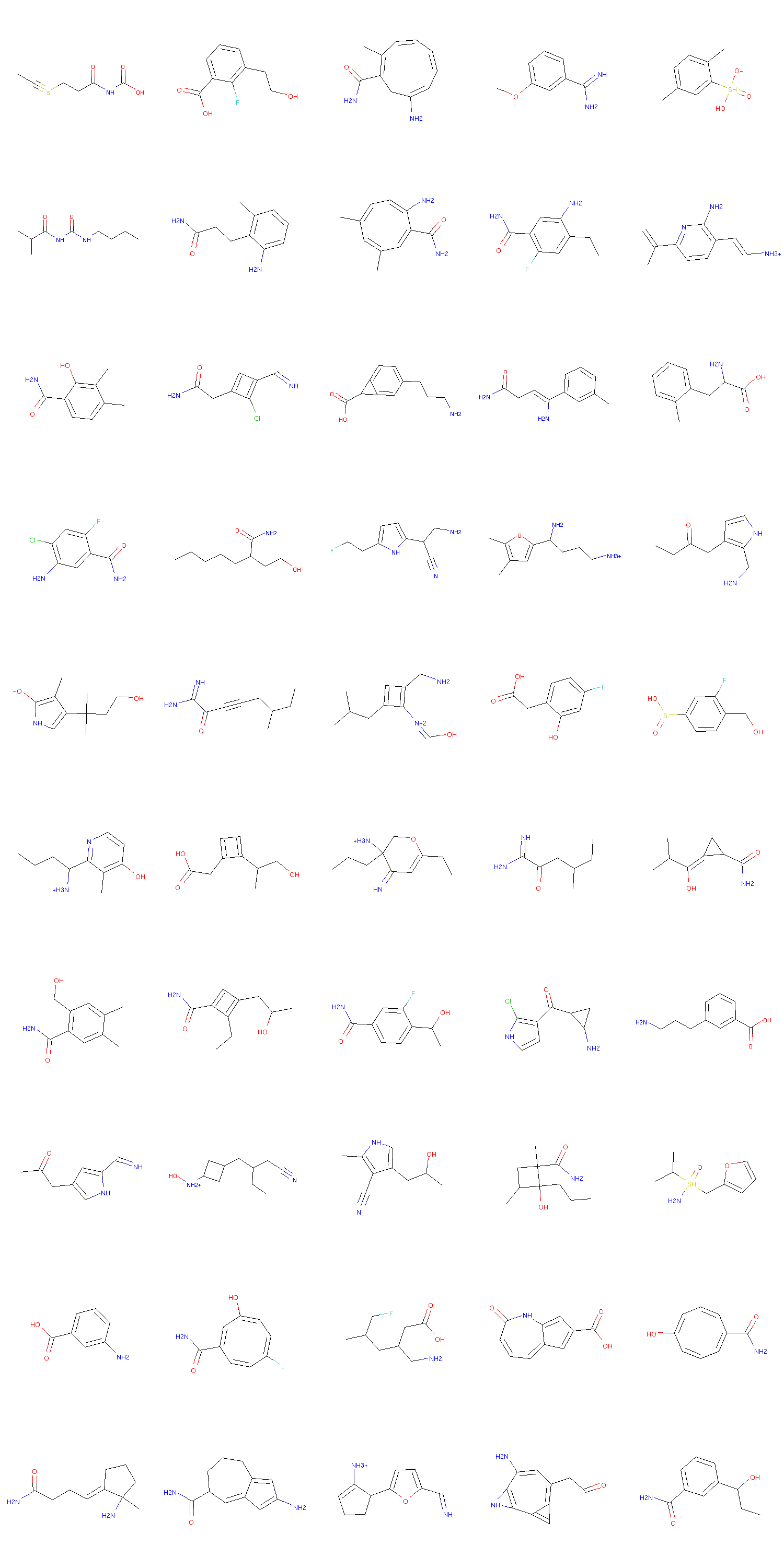

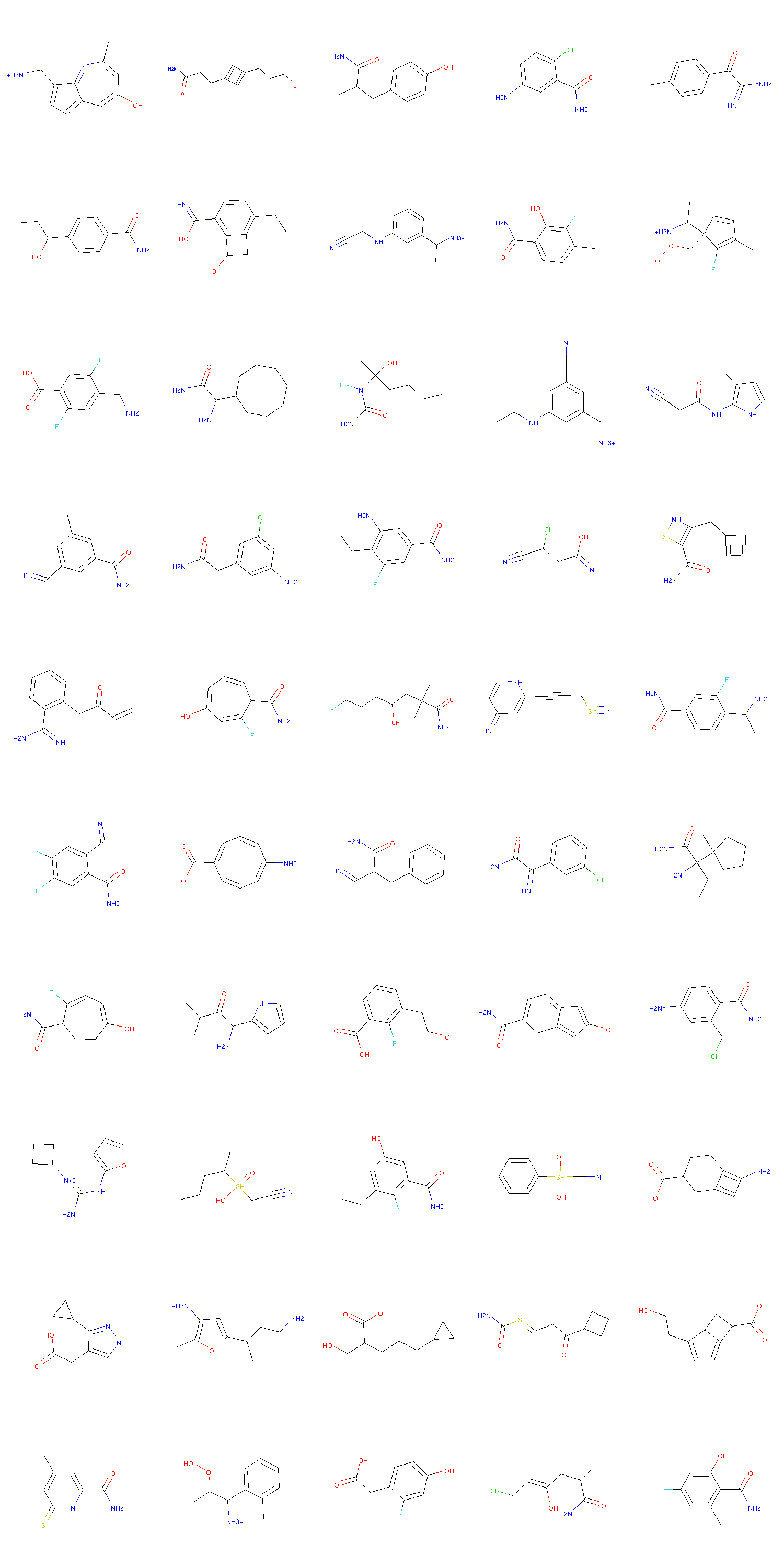


**Figure 5S**. 100 molecules whose 5 properties are same as those of Pregalalin
